# Radioprotective effect of *Rapana thomasiana* hemocyanin in gamma induced acute radiation syndrome

**DOI:** 10.1080/13102818.2014.924683

**Published:** 2014-07-10

**Authors:** Ivan Kindekov, Milka Mileva, Dimo Krastev, Vladimira Vassilieva, Yuliana Raynova, Lyuba Doumanova, Mitko Aljakov, Krassimira Idakieva

**Affiliations:** ^a^ Scientific Laboratory of Radiation Protection, Radiobiology and Cell Radiobiology, Military Medical Academy, Sofia, Bulgaria; ^b^ The Stephan Angeloff Institute of Microbiology, Bulgarian Academy of Sciences, Sofia, Bulgaria; ^c^ Department of Anatomy and Histology, College of Medicine ‘Yordanka Filaretova’, Sofia, Bulgaria; ^d^ Institute of Organic Chemistry with Centre of Phytochemistry, Bulgarian Academy of Sciences, Sofia, Bulgaria

**Keywords:** *Rapana thomasiana* hemocyanin, acute radiation syndrome, radioprotective effect, spleen colony assay, stomach ulcerations

## Abstract

The radioprotective effect of *Rapana thomasiana* hemocyanin (RtH) against radiation-induced injuries (stomach ulcers, survival time and endogenous haemopoiesis) and post-radiation recovery was investigated in male albino mice (C3H strain). Radiation course was in a dose of 7.5 Gy (LD 100/30 – dose that kills 100% of the mice at 30 days) from ^137^Cs with a dose of 2.05 Gy/min. Radiation injuries were manifested by inducing а hematopoietic form of acute radiation syndrome. RtH was administered intraperitoneally in a single dose of 50, 100, 150 and 200 mg/kg body weight (b. w.) once a day for five consecutive days before irradiation. The results obtained showed that radiation exposure led to (1) 100% mortality rate, (2) ulceration in the stomach mucosa and (3) decrease formation of spleen colonies as a marker of endogenous haemopoiesis. Administration of RtH at a dose of 200 mg/kg provided better protection against radiation-induced stomach ulceration, mitigated the lethal effects of radiation exposure and recovered endogenous haemopoiesis versus irradiated but not supplemented mice. It could be expected that RtH will find a use in mitigating radiation induced injury and enhanced radiorecovery.

## Introduction

Hemocyanins are copper-containing respiratory glycoproteins with quaternary structure. They are found in the haemolymph of some invertebrate species from the Molluska and Arthropod families. Hemocyanins are characterized with structural heterogeneity, high molecular weights and presence of carbohydrate component and act as strong activators of the immune system.[1,2] There are data showing that hemocyanins from different origin are widely used in laboratories and clinics as an immune stimulant and in the immunotherapy of bladder cancer and of renal cell carcinoma.[3] The possibility for protection of the organism after exposure to ionizing radiation, when the body absorbs high dose radiation energy, is practically nil.[4] Currently, in cases of accidental irradiation with high doses of radiation and developing of radiation syndrome, it is still possible to apply only conservative and symptomatic therapy.[5] A large number of drugs of synthetic and natural origin, e.g. antioxidants, cytoprotective agents, angiotensin-converting enzyme (ACE) inhibitors, etc., have been tested in both *in vitro* and *in vivo* models, and in human clinical trials to mitigate injuries caused by ionizing radiation exposure in sublethal and supralethal doses.[6–8]

The radioprotective effect of *Rapana thomasiana* hemocyanin (RtH) against radiation exposure has not been studied. The aim of the present work was to investigate the radioprotective effect of RtH administrated intraperitoneally at different single daily doses in mice irradiated by a lethal dose of 7.5 Gy.

## Materials and methods

### Preparation of hemocyanin

Native RtH was isolated from freshly obtained hemolymph of marine snails *Rapana thomasiana* by ultracentrifugation at 180,000×*g* (ultracentrifuge Beckman LM-80, rotor Ti 45) for 4 h at 4 °C and stored in the presence of 20% sucrose (w/v) at −20 °C until used. The purity of the isolated RtH was controlled by SDS- and native polyacrylamide gel electrophoresis (PAGE) as described previously.[9]

### Experimental animals and treatment

Male white mice C3H obtained from the Animal House of National Research, weighting about 22–25 g (8–10 weeks of age), were housed in cages with free access to drinking water and diet and maintained in the animal care facility throughout the duration of the experiment.

Experimental animals randomly divided into 10 experimental groups (*n* = 10) were placed in a specially designed well-ventilated acrylic container and the whole bodies of the animals were exposed to 7.5 Gy γ-irradiation (LD 100/30), given at a dose of 2.05 Gy/min from a ^137^Cs source, which produced a hematopoietic form of acute radiation syndrome. Propofol 100 mg/kg was administered intraperitoneally (*i.p*.) before the irradiation procedure.

Animal husbandry and the experiments were conducted in accordance with the guidelines of the Animal Care and Use Committee of the Bulgarian Academy of Science. The experimental groups are shown in Table 1.

**Table 1.** Experimental design of the study.

Experimental groups	RtH (mg/kg)
Non-irradiated mice	
Group I	0
Group II	50
Group III	100
Group IV	150
Group V	200
Irradiated mice	
Group VI	0
Group VII	50
Group VIII	100
Group IX	150
Group X	200

RtH was dissolved in phosphate-buffered saline (PBS) and administered intraperitoneally in doses of 50, 100, 150 and 200 mg/kg body weight (b. w.) for five consecutive days before irradiation. Up to day 30 after the radiation exposure animals were kept in the habitual conditions. On day 11 mice for spleen colony assay were anesthetized and sacrificed by decapitation.

### Histological study

The procedure for preparing preparations for histological study was according to the standard protocol approved by the Department of Anatomy and Histology of Medical University, Sofia. Macroscopic and microscopic evaluation and histological analyses of ulcers were performed. The stomachs were immersed in 4% neutral formalin, and seven days later a procedure of dehydration in alcohol was performed, following lightening in cedar oil. Then, the samples were embedded in paraffin, and series of cuts 20 μm in thickness were prepared.

The stomachs were collected on the 4th, 12th and 24th hour after irradiation and filled with 1.0% formalin for 10 min. The mucose were exposed by opening the stomachs along the greater curvature. The length and the width (mm) of ulcers on the gastric mucosa were measured with a planimeter (1 mm × 1 mm) under a dissecting microscope (×0.7–3.0; Carl Zeiss). The ulcer areas were determined as described by [10]
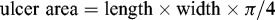



The total ulcer area in square millimetres (mm^2^) was scored.

### Spleen colony assay

On day 11 following irradiation mice for spleen colony assay were put to death through cervical dislocation. Their spleens were extracted and fixed with Bouin solution for 24 h, then laved with 86% alcohol. Proliferated colonies on the parietal surface of the spleen that were over 0.2 mm in diameter were counted three times and the mean number of colonies was taken into account.[11]

### Antioxidant properties of RtH in liposomal suspension

We used a liposomal suspension obtained from phospholipids of egg yolk extracted according to Folch et al.[12] After evaporation under vacuum, the chloroform fraction was dissolved in 50 mmol/L K-Na phosphate buffer, pH 7.4, to a final concentration of lipids of 2 mg/mL.

Antioxidant activity (AOA) of RtH in liposomal suspension was measured by formation of endogenous lipid peroxidation products reacting with 2-thiobarbituric acid (TBARS), and detected spectrophotometrically (λ_max_ = 532 nm) as described by Mileva et al.[13] The induction of lipid peroxidation was initiated by adding 50 μL of Fe^2+^ to a final concentration of 1 mmol/L. Each sample contained 1.8 mL of liposomal suspension with lipid concentration of 1 mg/mL, 50 μL of 2.1 mmol/L ascorbic acid and 100 μL solution of RtH to obtain concentrations of 10, 50, 100 and 200 μg/mL. The amount of TBARS generated in the system was determined after incubation for 30 min at 37 °C. The activity of RtH was compared against the activity of butyl hydroxytoluene (BHT), a common antioxidant, at the same concentrations. The AOA was defined as the ratio of the absorption at 560 nm for the sample containing the tested substances in different concentrations and the same absorption for the controls (without RtH or BHT) expressed in percentage terms.

All data were presented as means ± standard error (SE) and analysed using one-way analysis of variance (ANOVA). Survival rate was evaluated by the Kaplan–Meier test (SPSS for Windows, ver.16).

## Results and discussion

In the present study we investigated an aspect of the biological activities of RtH – its radioprotective capability in an experimental mice model after supplementation with daily doses of 50 mg/kg b. w to 200 mg/kg b. w for 5 consecutive days before radiation exposure.

The administration of different doses of RtH for five consecutive days did not induce morbidity and mortality. Mortality was observed for 30 consecutive days after the last day of administration. Irradiation of animals with 7.5 Gy resulted in sickness within 7–10 days after exposure. The symptoms included reduction in the food and water intake, weight loss, diarrhoea. All of them are reported as gastrointestinal and hematopoietic symptoms.[7,14] In our experiments, doses of 50 and 100 mg/kg b. w. of RtH had similar effects on the survival rate after the radiation damage. Pretreatment with RtH in doses of 150 and 200 mg/kg b. w. led to an increase in the period (up to 10 days) without clinical symptoms induced by radiation.

Evaluation of 30-day survival rate after lethal whole body irradiation is showed as a gold standard for radioprotective activity.[6] The results demonstrating the radioprotective potential of RtH on the survival rate of mice are shown in Table 2. Exposure to ionizing radiation with a dose of 7.5 Gy caused 100% mortality for the whole period. Death occurred between the day 10 and day 15 after radiation exposure.

**Table 2.** Effect of RtH on the survival rate of experimental mice supplemented with different doses of RtH for five consecutive days before exposure to gamma irradiation with a dose of 7.5 Gy. The experimental groups are as described in Table 1.

		Mortality on different days post-treatment							
Groups	RtH (mg/kg b. w.)	5	10	15	20	25	30	Mortality (%)	Survivors/total
Non-irradiated mice									
I	0							0%	10/10
II	50							0%	10/10
III	100							0%	10/10
IV	150							0%	10/10
V	200							0%	10/10
Irradiated mice									
VI	0		3	7				100%	0/10
VII	50			5	3			80%	2/10
VIII	100			5	2			70%	3/10
IX	150		3			1		40%	6/10
X	200			5				50%	5/10

**Figure 1.**
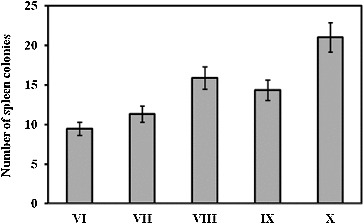
Spleen colony assay on day 11 post-irradiation. The experimental groups are designated as described in Table 1.

Spleen colony assay was performed on day 11 after irradiation. The results are shown in Figure 1. The administration of RtH in a dose of 200 mg/kg b. w. increased the number of endogenous spleen colonies in the experimental animals in group X as compared to that in the irradiated, but not supplemented mice in group VI (*p* < 0.05). The spleen colony assay showed that when the mice had received lethal irradiation to suppress endogenous haemopoiesis, on day 11 after irradiation endogenous colonies were produced in the spleen (Figure 1). These colonies were from the pluripotent cells, which derived from the reserved cells in the bones of irradiated animals. The colonies varied in morphology: erythroid, granulocyte or mixed. The nodules observed in the spleens of the irradiated mice are usually discrete, round or oval, grey in colour, and embedded in the red mass of the spleen.[15,16] Colony-forming units are very sensitive to radiation influence.[17,18] These cells are indicative of regeneration capabilities of haematopoiesis. The formation of haematopoietic colonies in the spleen after irradiation is thought to be a function of surviving pluripotent stem cells, i.e. cells which respond to an appropriate stimulus by differentiation into red cells, white cells, or platelets and which are capable of self-replication.[19] The results of the spleen colonies test showed that regeneration of the stem cells after radiation exposure followed a wavy line, and reached its maximum between the 2nd and the 5th hour, decreased to the 11th hour and maintained steady values between the 20th and the 25th hour after irradiation.[4,8,11] Cell regeneration typically occurs before the resumption of the suppressed mitotic activity of the cells.[15,16,20] RtH was applied at a prophylaxis regimen before the radiation exposure. The results suggest that this scheme may be the reason for the lack of stem cells line regeneration. We suppose that there could be several aspects for the protective action of the preparation: (1) provocation of the cells reparative processes, (2) stimulation of the stem cells pools by differentiation, (3) activation of the antioxidant defence, as well as (4) impact on the medullar microenvironment.

**Figure 2.**
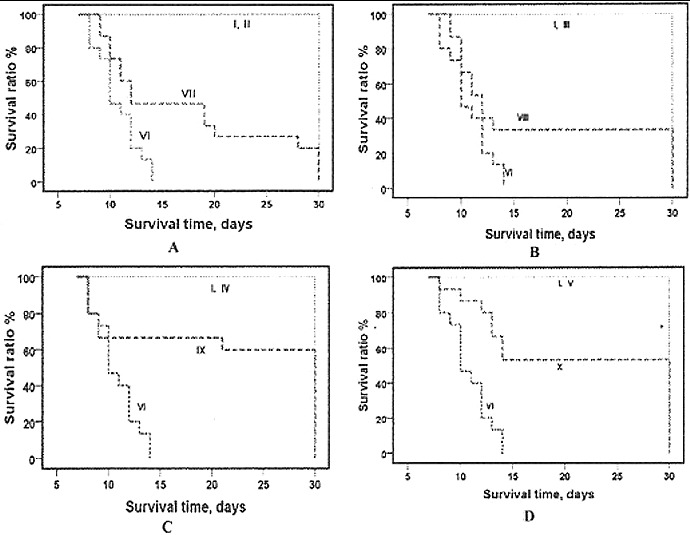
Survival curves of mice supplemented with different doses of RtH: 50 mg/kg (A), 100 mg/kg (B), 150 mg/kg (C) and 200 mg/kg (D). The experimental groups are designated as described in Table 1. Mice were irradiated with 7.5 Gy gamma radiation. Survival was monitored up to 30 days post-irradiation. Statistically significant difference between Group VI (irradiated only), and irradiated Groups supplemented with RtH ( *p* < 0.05).

The administration of RtH *i.p.* for five consecutive days increased the life expectancy of the experimental animals (Figure 2). The survival curves showed that the point of early death was shifted to the right: between day 11 to day 15 of exposure and administration with 50, 100, 150 and 200 mg/kg b. w. of RtH. The statistical analysis of the results showed significant difference between the post-irradiation survivals in the group which was administered RtH versus the irradiated only group, and versus the non-irradiated group supplemented with different doses of RtH. The prolongation effect of the doses of 150 RtH and 200 mg RtH on the mortality can be seen clearly in Figure 2(C) and 2(D).

**Figure 3.**
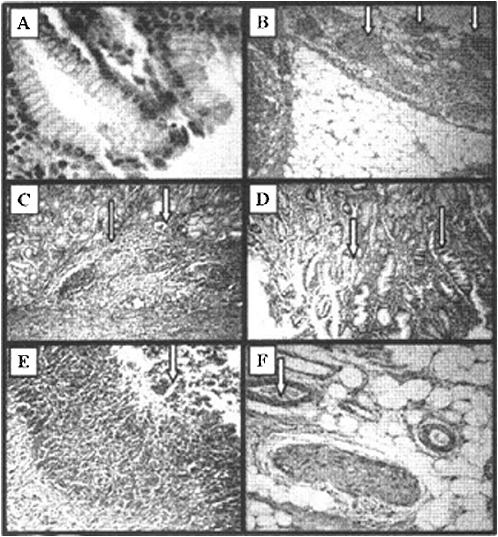
Histological haematoxylin/eosin (HE) analysis of experimental gastric ulcers induced by gamma irradiation. Stomach of healthy mice (A), HE × 10; nerve endings in the underlying tissue of the stomach in healthy mice (B), HE × 40; ulceration on the 4th hour after irradiation (C) – noduli lymphatici (thin arrow) and dilated vessel (thick arrow), HE × 40; and vacuolization (D) of the stomach epithelium (thin arrow) and area of normal epithelium (thick arrow), HE × 40; stomach integrity on the 12th hour (E) and 24th hour (F) after radiation exposure; HE × 40.

Our study was focused on the stomach ulcers as a morphological marker of gastrointestinal radiation injuries.[7] The same changes could be observed during the hematopoietic form of acute radiation syndrome.[4] Microscopic observation showed that γ-irradiation induced gastric mucosal lesions (Figure 3). Histological haematoxylin/eosin (HE) analysis of experimental gastric ulcers induced by gamma irradiation showed that the ulceration started at the 4th hour after irradiation (Figure 3(C) and 3(D)). The small light vacuoles appearing in the cytoplasm are additional extensions of smooth endoplasmic reticulum. Figure 3(C) shows noduli lymphatici (thin arrow) and dilated vessel (thick arrow), and Figure 3(D) – vacuolization of the stomach epithelium (thin arrow) and area of normal epithelium (thick arrow). Stomach integrity on the 12th and 24th hour (Figure 3(E) and 3(F)) after radiation exposure was disturbed, as manifested by the presence of a large amount of purulent exudate (thick arrow) composed of neutrophils, necrotic cells and edematous fluid.

**Figure 4.**
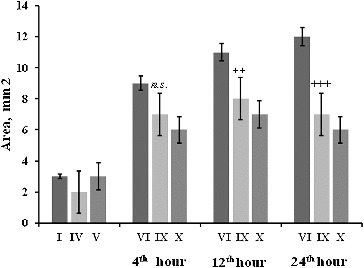
Stomach ulcer area (mm^2^) of mice 4–24 h after irradiation exposure. The experimental groups are designated as described in Table 1. Statistical significance: *** *p* < 0.001 vs. Group I; +++ *p* < 0.001 vs. Group VI; ++ *p* < 0.01 vs. Group VI; *n.s.* – non-significant.

Macroscopic observation showed that the stomach ulcer area (Figure 4) was increased at the 4th, 12th and 24th hour post-irradiation. The changes in the stomach mucosa at the 4th hour showed that radiation induced stress ulcerations on the stomach mucosa of mice. RtH in a dose of 200 mg/kg mitigated the effect of the radiation factor. On the 12th and 24th hour post-irradiation, the mitigation effect increased in a dose-dependent manner. Lower doses of RtH did not have a pronounced protection effect on the stomach mucosa (Figure 4).

Gastric ulcers often result from an acute single exposure of total body irradiation, which suggests that the ulcers produced in our experimental conditions were probably acute peptic acid ulcers, and since the animals died shortly thereafter, there was no opportunity for healing or for the development of delayed injury.[21] Since these were all early experimental ulcers, they could most likely be attributed to direct radiation-induced mucosal cell damage. Capillary endothelial cell abnormalities and acute thromboses may have added injurious ischemic effects.[22]

**Figure 5.**
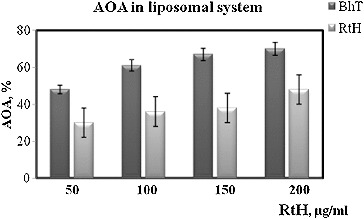
Antioxidant activity (AOA) of RtH and BHT in liposomal syspension. Data are expressed as mean ± SE.

Gastric mucosal integrity is maintained by a dynamic process of cell death and cell proliferation.[23] The mechanism of formation of gastric mucosal lesions is not well studied but oxidative damages and apoptotic cell death, along with other factors, are generally considered to be involved in the loss of gastric mucosal integrity.[24] There data show that a series of events happen in cells: (1) free radicals are generated, (2) lipid peroxidation takes place and (3) apoptotic processes develop. Reactive oxygen species are known to play a role in the induction and pathogenesis of gastric injury,[25] and lesions develop when oxidative damage and apoptosis dominate over the healing process.[18] That is why we assumed that RtH may possesses some antioxidant properties. It is known that the change in the level of production of free radicals in the organism changes the level of the lipid peroxidation as well. Therefore, in our subsequent experiments we checked the AOA of RtH as compared to a common antioxidant BHT in an experimental liposomal system. RtH could possibly exhibit radical-scavenging properties, and this effect is probably connected with mitigation of radiation injuries. For a more detailed analysis of the mechanism of the radioprotective effect of RtH a more complete study of its antioxidant profile is necessary. Figure 5 shows the results of Fe^2+^-induced oxidation of an aqueous emulsion system of egg liposomes as an antioxidative activity test of RtH in the concentration range of 10–200 μg/mL. The results are expressed as percentage of inhibition of the oxidation process in comparison to control sample (without tested substance). Our findings demonstrated that RtH had a lower AOA versus BHT. For all concentrations tested AOA of RtH was about 30% less than that of BHT.

A review of the scientific literature shows that a radioprotective agent can be defined as ‘any agent that protects against radiation-induced damage, whether administered before, during, or after irradiation’.[20] They have been classified into three categories: (1) prophylactic agents, (2) mitigators and (3) therapeutic agents.[18] Prophylactic agents are administered before radiation exposure to prevent damage. Radioprotectors function on the premise that some chemicals when given before irradiation protect the vital biomolecules from radiation-induced lesions, either by preventing the initial damage to the vital tissues or by restituting the original structure by repair, or both.[20,26,27] The modern approach of understanding the mechanism of the antiulcer effect of protective compounds such as RtH should therefore be directed towards exploring its possible role in preventing oxidative damage and apoptosis as well as on the promotion of healing process by cell proliferation.[26] A radioprotective agent should protect normal tissues that are considered sensitive, should reduce acute or late toxicities in these tissues and finally should be responsible for a significant improvement in the quality of life, not only symptomatically but also on the functional level (i.e. mucositis, haematopoiesis, etc.). The results of this study demonstrate that the administration of RtH in a dose of 200 mg/kg b. w. mitigates the lethal effects of radiation exposure. RtH possesses a number of biological properties that may contribute to its radioprotective efficacy.

## Conclusions

In our experiments, RtH showed a trend to mitigate the lethal effects of radiation. Our results clearly indicated the capacity of RtH in these experimental conditions to modulate the recovery and regeneration of the gastrointestinal epithelium as well as the hematopoietic progenitor cells in the bone marrow, which are the two most radiosensitive organs that are essential for the survival of the irradiated body. 
